# Expansion of Intrinsically Disordered Proteins Increases the Range of Stability of Liquid–Liquid Phase Separation

**DOI:** 10.3390/molecules25204705

**Published:** 2020-10-15

**Authors:** Adiran Garaizar, Ignacio Sanchez-Burgos, Rosana Collepardo-Guevara, Jorge R. Espinosa

**Affiliations:** 1 Maxwell Centre, Cavendish Laboratory, Department of Physics, University of Cambridge, J J Thomson Avenue, Cambridge CB3 0HE, UK; ag949@cam.ac.uk (A.G.); is490@cam.ac.uk (I.S.-B.); rc597@cam.ac.uk (R.C.-G.); 2 Department of Chemistry, University of Cambridge, Lensfield Road, Cambridge CB2 1EW, UK; 3 Department of Genetics, University of Cambridge, Downing Site, Cambridge CB2 3EJ, UK

**Keywords:** proteins, biological phase transitions, computer simulations

## Abstract

Proteins containing intrinsically disordered regions (IDRs) are ubiquitous within biomolecular condensates, which are liquid-like compartments within cells formed through liquid–liquid phase separation (LLPS). The sequence of amino acids of a protein encodes its phase behaviour, not only by establishing the patterning and chemical nature (e.g., hydrophobic, polar, charged) of the various binding sites that facilitate multivalent interactions, but also by dictating the protein conformational dynamics. Besides behaving as random coils, IDRs can exhibit a wide-range of structural behaviours, including conformational switching, where they transition between alternate conformational ensembles. Using Molecular Dynamics simulations of a minimal coarse-grained model for IDRs, we show that the role of protein conformation has a non-trivial effect in the liquid–liquid phase behaviour of IDRs. When an IDR transitions to a conformational ensemble enriched in disordered extended states, LLPS is enhanced. In contrast, IDRs that switch to ensembles that preferentially sample more compact and structured states show inhibited LLPS. This occurs because extended and disordered protein conformations facilitate LLPS-stabilising multivalent protein–protein interactions by reducing steric hindrance; thereby, such conformations maximize the molecular connectivity of the condensed liquid network. Extended protein configurations promote phase separation regardless of whether LLPS is driven by homotypic and/or heterotypic protein–protein interactions. This study sheds light on the link between the dynamic conformational plasticity of IDRs and their liquid–liquid phase behaviour.

## 1. Introduction

The cell interior contains thousands of different biomolecules (e.g., multivalent proteins and RNAs), which need to be organized in space for the cell to function. One of the key cellular mechanisms to control the spatial organization of components is formation and dissolution of biomolecular condensates—membraneless compartments sustained by the physical chemistry of liquid–liquid phase separation (LLPS) [1,2,3].

Although the concept of liquid-like membraneless compartments inside cells [4,5] is not new (it is attributed to Edmund Wilson in the 19th century [6]), emerging evidence on the wide-ranging roles that biomolecular condensates play inside cells has reignited interest in the phenomenon of biological LLPS (see reviews: [2,7,8]). Besides compartmentalisation of the cytoplasm [2,8,9,10,11,12], LLPS is involved in genome silencing [13,14,15], the formation of pathological aggregates such as amyloid fibrils [3,16,17,18], and helping cells to sense and react to environmental changes [19]. Novel biological features, such as the ability of condensates to buffer protein concentrations against gene expression noise [20] continue being discovered.

Many proteins containing intrinsically disordered regions (IDRs) such as FUS [21,22,23], hnRNPA1 [24,25], TDP-43 [26,27], and HP1 undergo LLPS in vitro and in cells [14,15]. IDRs that contribute to phase separation preferentially establish multiple homotypic self-interactions or heterotypic interactions with a cognate biomolecule (e.g., a different IDR or RNA) over their interactions with the solvent [28,29,30]. Such ability of biomolecules to establish multiple transient interactions is known as multivalency [31,32,33]. Due to their conformational versatility, IDRs can also promote LLPS by acting as inert but highly flexible linkers that connect modular domains [34,35].

The structural and dynamic behaviour of IDRs varies widely. IDRs can behave as random coils [36,37], exhibit disorder-to-order structural transitions [38,39,40,41,42], and even switch among different conformational ensembles [40,43,44,45]. Conformational switching describes a behaviour in which IDRs can transition between alternate conformational ensembles (free energy valleys of their conformational landscape) where their structure, despite remaining partially disordered, fluctuates respect to an equilibrium conformation [46,47]. The conformational switching behaviour of IDRs can be regulated by many different external events, such as changes in the physiological conditions as ion influx [48], or introduction of post-translational modifications such as phosphorylation [49,50,51]. Also, spontaneous switching among different conformational ensembles has been reported for the C-terminal tail of the GluN2B subunit of the N-methyl-D-aspartate receptor [52], where post-translational modifications or fluctuations in the ion concentration and/or pH drive the IDRs to switch between alternate conformational ensembles.

Experimental advances in single molecule Förster resonance energy transfer (smFRET) have now enabled the direct observation of conformational switching in diluted conditions [53,54,55]. However, how conformational switching and the role of IDR conformation impacts the liquid–liquid phase behaviour of IDRs is not fully understood. Computer simulations offer a complimentary technique to investigate this question because they can resolve the structural behaviour of individual interacting proteins as they undergo LLPS, while quantifying changes in critical parameters.

Atomistic Molecular Dynamics (MD) simulations can characterize the conformational ensembles of single proteins and protein complexes [56,57,58], pinpoint the link between chemical modifications and sequence mutations, and the modulation of protein–protein and protein-DNA interactions [59,60,61,62], reveal the conformational heterogeneity of IDRs within small aggregates [63], and guide the development of chemically accurate coarse-grained models for LLPS [64,65]. Furthermore, the predictive and explanatory power of atomistic simulations is constantly being ramped up by the collective efforts to develop even more accurate atomistic force fields for IDRs [66,67].

Powered by algorithmic advances and increased computer capabilities, atomistic MD simulations are now being used to investigate protein interactions within crowded environments that mimic in vivo conditions [68]. Simultaneously, a wide-range of coarse-grained (CG) models, including mean field, thermodynamic perturbation theory, lattice-based representations [28,69,70,71,72,73,74,75,76,77], minimal models [10,11,78,79,80,81,82,83] and sequence-dependent chemically accurate CG models [64,84,85], are being developed to investigate the link between microscopic protein characteristics and their phase behaviour. Coarse-grained models have the ability to retain specific physico-chemical features of proteins and investigate how they might impact their phase behaviour, while averaging out others for computational efficiency. As such, coarse-grained models were successful at identifying the dependency of biomolecular condensate stability on the protein chain length [86,87], amino acid sequence [75,84,85,88,89], protein multivalency [34,90,91,92,93], topology [94] and multi-component composition [10,95,96,97].

In this work, using MD simulations of a minimal coarse-grained protein model, we investigate the impact of modulating the conformational landscape of IDRs on their ability to form biomolecular condensates. Our results show that conformational switching enhances LLPS whenever the IDR configurational landscape is enriched in extended states. Consistently, exploring preferentially more compact and structured globular states (such as after a disorder-to-order transition) hinders LLPS by limiting the protein valency. We find that these observations are explained by a larger density of molecular connections that extended IDRs can establish within the condensed liquid. These results illustrate how beyond the amino acid sequence, the connectivity of the condensed liquid network, which governs the ability of proteins to phase separate [10], crucially depends also on the conformational ensemble that an IDR adopts. Moreover, we find that this behaviour holds regardless of whether protein LLPS is driven by homotypic and/or heterotypic interactions. Taken together, our observations shed light on the connection between the conformational plasticity of IDRs and their phase behaviour.

## 2. Results and Discussion

### 2.1. A Minimal Coarse-Grained Model for IDRs

**Figure 1:**
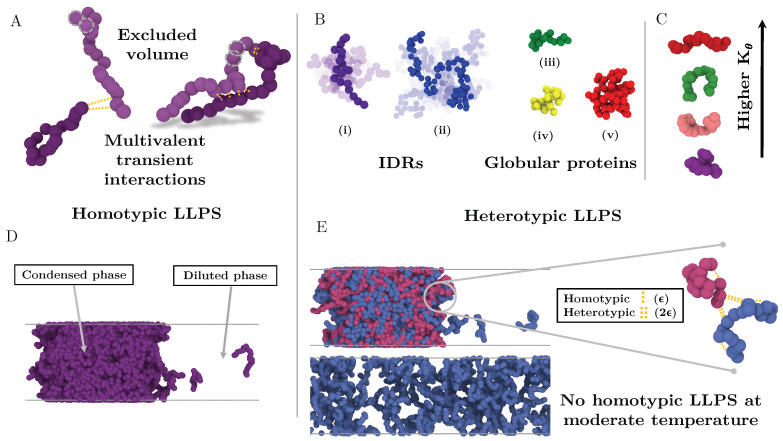
Minimal IDR coarse-grained model to investigate the impact of the conformational ensemble in LLPS. (**A**) Illustration of the coarse-grained protein model developed in this work with one bead representing a group of amino acids. The excluded volume interactions among beads are depicted with grey dashed circles, and the multivalent attracting interactions with yellow dashed lines. (**B**) Representative snapshots of the different types of IDRs studied. (i) Intrinsically disordered protein of N = 20. (ii) Intrinsically disordered protein of N = 80. (iii) Semi-compact globular protein of N = 20. (iv) Compact globular protein of N = 20. (v) Compact globular protein of N = 80. (**C**) Schematic representation of the impact of the angular constant ($K_{\theta }$) in the representative configuration of the coarse-grained IDRs of N = 20. Higher $K_{\theta }$ implies conformational landscapes enriched in extended states while lower ones enhance the emergence of structures preferentially collapsed. (**D**) Snapshot of a direct coexistence simulation in which LLPS is driven by homotypic interactions. (**E**) Sketch of a binary mixture direct coexistence simulation where LLPS is driven by heterotypic interactions, phase separation takes place in presence of a high affinity partner. Multivalent interactions between proteins of the different type (heterotypic) are twice more attractive (2ϵ) than those among proteins of the same type (ϵ).

Based on the ability of IDRs to exhibit large conformational fluctuations [29,30] and the dominant role of multivalency in LLPS [10,98], we develop a minimal coarse grained model that allows us to modulate the structural plasticity of multivalent IDRs. In our model, each IDR consists of a flexible polymer of *N* beads, where each bead represents a group of several amino acids (see Figure 1).

We capture the ability of phase-separating IDRs to establish numerous weak and promiscuous protein–protein interactions at short molecular distances, with a short-ranged attractive Lennard-Jones potential among protein beads:

(1) $$u_{\mathrm{LJ}}=4\epsilon \left[{\left(\frac{\sigma }{r}\right)}^{12}-{\left(\frac{\sigma }{r}\right)}^{6}\right]$$

where σ accounts for the molecular diameter of each bead, *r* is the inter-bead distance, and ϵ defines the maximum attractive interaction among different beads. In what follows, σ is used as the unit of length and ϵ as the unit of energy. This potential approximates the various types of protein–protein interactions driving LLPS (e.g., hydrophobic, electrostatic, cation-π, π-π). To account for the covalent bonds among sequential groups of amino acids within a single IDR, consecutive beads are joined together with a stiff harmonic potential, $u_{\mathrm{Bond}}$, of the form:

(2) $$u_{\mathrm{Bond}}=K_{\mathrm{Bond}}{\left(r-r_{0}\right)}^{2},$$

where $K_{\mathrm{Bond}}$ controls the stiffness of the bond and $r_{0}$ is the equilibrium bond length. Additionally, we introduce an harmonic angular potential to modulate the conformational plasticity of IDRs:

(3) $$u_{\mathrm{Bend}}=K_{\theta }{\left(\theta -\theta_{0}\right)}^{2},$$

where $K_{\theta }$ controls the strength of the bending potential, θ defines the angle formed by three consecutive beads, and $\theta_{0}$ represents the equilibrium resting angle ($\theta_{0}=180{}^{\circ}$). Non-bonded interactions between beads directly bonded to each other are excluded. For computational efficiency, solvent is represented implicitly; hence the protein-poor liquid phase corresponds to a vapour and the protein-rich liquid phase (condensate) to a liquid phase.

### 2.2. Simulation Details

For convenience, we express the following magnitudes in reduced units: temperature as $T^{*}$ = $k_{B}T/\epsilon$, number density as $\rho^{*}$ = (N/V)$\sigma^{3}$, pressure as $p^{*}$ = $p\sigma^{3}/\epsilon$, time as $\sigma \sqrt{m/\epsilon }$, and the angular constant as $K_{\theta }^{*}$ = $K_{\theta }rad^{2}/\epsilon$, being $\epsilon /k_{B}$ = 119.81 K, σ = 3.405 and *m*, the mass of a single bead, 39.1 amu. These values were chosen so that the temperatures and densities at which liquid–liquid phase behaviour occurs in the model are realistic in real units (i.e., from 250 to 350 K and from 0.4 to 1.2 g/cm${}^{3}$ respectively.)

**Figure 2:**
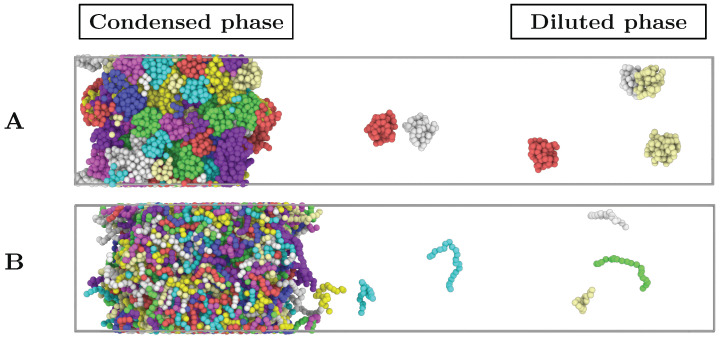
Direct coexistence simulation box of: (**A**) 128 interacting globular proteins of length N = 80 at $T^{*}=2.5$. (**B**) 512 intrinsically disordered proteins of length N = 20 at $T^{*}=2.25$. Individual proteins are depicted by different colours.

The parameters for the bonded interactions in Equation (2) are $K_{Bond}$ = 40 $\epsilon /\sigma^{2}$, and the equilibrium bond length $r_{0}=1\sigma$. All our simulations were performed in the MD package LAMMPS [99]. The integration timestep used to numerically solve the equations of motion is $\Delta t^{*}=0.0004$. For NVT and NpT simulations, we employ the Nosé-Hoover thermostat and barostat [100,101] with relaxation times of $\Delta t^{*}=0.4$ and $\Delta t^{*}=0.401$ respectively. The cut-off for the LJ interactions is set to 2.5 σ. To maintain the structure of the globular domains, we use the LAMMPS rigid body integrator [102]. The typical system size for simulations of 20-bead IDRs is 512 replicas, while for those of 80-bead IDRs is 128. To minimise finite system size effects in Direct Coexistence (DC) simulations [103,104,105] (see Section 3 and Figure 2) and ensure accurate predictions close to the critical point, we repeat simulations at high temperatures with 1024 protein replicas for IDRs of N = 20 and with 256 replicas for IDRs of N = 80.

### 2.3. LLPS Is Promoted by Extended IDR Configurations

As mentioned in the introduction, IDRs span a wide-range of different structural behaviours, including acting as fully disordered random coils [36,37], serving as flexible linkers [34,35] between globular domains, exhibiting disorder-to-order structural transitions [38,39,40,41,42,106] or even switching between alternate conformational free energy minima while remaining partially disordered [46,47]. In this section, we use our minimal CG model to investigate how the phase behaviour of IDRs changes depending on their conformational landscape. For this, we compare the phase diagrams, in temperature-density space, of an IDR of fixed amino acid sequence when it behaves as a random coil versus when it transitions to sample an energy landscape relatively enriched in more extended or more collapsed conformations.

**Figure 3:**
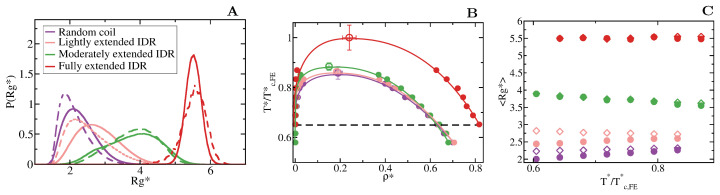
Conformational landscape and phase diagram of the different 20-bead IDRs. (**A**) Probability histograms of the radius of gyration of the different IDRs at $T^{*}=0.65T_{c,FE}^{*}$. $T_{c,FE}^{*}$ refers to the critical temperature of the fully extended IDR, $T_{c,FE}^{*}$ = 2.76, which is the highest critical temperature of the four different IDRs. Results for the fully flexible random coil ($K_{\theta }^{*}$ = 0) are depicted in purple, while those ones for the lightly ($K_{\theta }^{*}$ = 1), moderately ($K_{\theta }^{*}$ = 3) and fully extended ($K_{\theta }^{*}$ = 20) IDRs in pink, green and red, respectively. Solid lines represent the radius of gyration distribution for proteins that form part of the condensed liquid phase, whereas dashed lines account for the radius of gyration distribution of proteins belonging to the diluted liquid phase. (**B**) Liquid-liquid coexistence lines in the $T^{*}-\rho^{*}$ plane for the fully flexible random coil (purple), lightly extended (pink), moderately extended (green) and fully extended (red) IDRs. The temperature is normalized by the critical temperature of the fully extended IDR, $T_{c,FE}^{*}$. Filled circles account for the coexisting densities computed via DC simulations and empty ones for the estimation of the critical points using Equations (4) and (5). The horizontal black dashed line represents the temperature at which the radius of gyration probability histograms in panel A were evaluated. (**C**) Mean value of the radius of gyration ($<R_{g}^{*}>$) as a function of the renormalized temperature, $T^{*}/T_{c,FE}^{*}$, for the previously shown IDRs. The same colour code as in panel A and B applies here. The values of $R_{g}^{*}$ in the condensed phase along the coexisting densities are depicted by empty diamonds whereas filled circles represent the same but for IDRs in the protein-poor liquid phase.

We first focus on IDRs whose LLPS is driven by homotypic interactions. To exclude amino acid sequence and patterning effects [84,85,89], we maintain the IDR amino acid sequence constant by modelling self-interacting IDRs as homopolymers with a constant value of ϵ and σ for all the beads (see Equation (1)). IDRs behaving as random coils are represented as fully flexible chains (i.e., without an energetic penalty for bending, $K_{\theta }^{*}$ = 0), while IDRs that have undergone conformational switching to sample a landscape enriched in more extended configurations are modelled by gradually increasing the energetic penalty for bending. Therefore, $K_{\theta }^{*}$ allows us to mimic different conformational free energy minima that IDRs exhibiting conformational switching may have. In Figure 3A, we plot the probability histograms of the radius of gyration ($R_{g}^{*}$) for 20-bead IDRs that present four different structural behaviours: a fully flexible random coil (purple), a lightly extended IDR (pink), a moderately extended IDR (green), and a fully extended IDR (red). The wide distributions of the radii of gyration evidence that the four alternate ensembles sampled by the IDRs are highly heterogeneous. That is because all proteins retain a high degree of flexibility and remain fully disordered in all cases. The moderately extended IDR shows the widest variation in the radius of gyration due to the interplay between the attractive intra-molecular interactions that favours compaction and a moderate energetic penalty for bending that opposes it. The fully extended IDR exhibits the narrowest variation in the radius of gyration among the set, consistent with its conformational ensemble being highly shifted towards extended states.

To investigate the impact of the conformational behaviour of IDRs in their ability to phase separate, we use DC simulations (Figure 2 and Section 3) to compute the phase diagrams of the different IDRs. We find that for a constant amino acid sequence, the liquid–liquid coexistence region becomes larger when the IDR exhibits a conformational ensemble that is more enriched in extended states (Figure 3B). These results are in agreement with short linear and rigid LJ polymers of 3 to 5 beads shown to display a significantly wider liquid-vapour coexistence region than their flexible counterparts [107]. Nonetheless, the opposite behaviour (i.e., semiflexible polymers being less prone to phase separate than flexible ones) was observed for polymer chains with bond angles of $109.5^{\circ }$ [108], suggesting, that stiffness only promotes two-phase demixing when such rigidification results in more extended polymer conformations. That rigidification of extended IDR states that increases the stability of their condensates may also help explain what drives the liquid-gel transitions which are implicated in the formation of pathological aggregates [3,16,17,18]. Moreover, subtle effects in polymer stiffness and conformation has also been shown to drive demixing of polymer mixtures into two-phase equilibrium [109].

**Figure 4:**
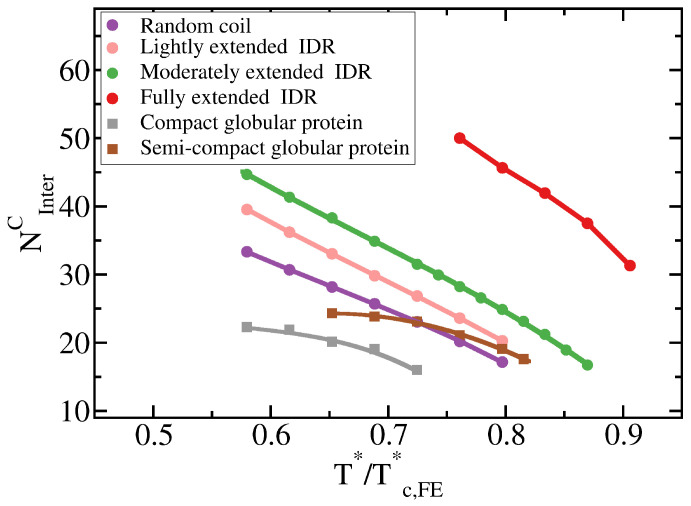
Liquid-network connectivity explains LLPS. Average number of inter-molecular contacts per protein in the condensed liquid phase, $N_{Inter}^{C}$, as a function of $T^{*}/T_{c,FE}^{*}$ for the different 20-bead proteins described in the legend.

To further understand this observation, we evaluate the molecular connectivity (further details in Section 3.2) within the condensed-liquid network for each IDR type by computing the number of inter-molecular contacts per protein replica [10]. More extended IDR configurations promote a significantly higher number of inter-molecular contacts within the condensed phase. The larger number of molecular contacts that fully extended IDRs can establish in comparison to the relative more compact random coils or lightly extended IDRs (see Table 1 and Figure 4), stabilises the protein condensates up to higher temperatures. Therefore, the conformational ensemble of an IDR impacts its liquid–liquid phase behaviour by directly regulating the number of inter-molecular contacts (liquid-network connectivity [10]) that an IDR can establish within the condensed liquid.

When we evaluate the mean radius of gyration ($<R_{g}^{*}>$) of the different IDRs as a function of temperature and compare values when the IDRs are part of the diluted phase or part of the condensed phase (see Figure 3C), we observe that the conformational landscape of an IDR has a non trivial effect on their temperature-dependent behaviour. Fully flexible random coils and lightly extended IDRs undergo temperature-induced expansion while IDRs more significantly constrained to sample extended states exhibit temperature-induced collapse. In contrast, the mean value of the radius of gyration of fully extended IDRs remains constant for the whole range of temperatures both within the condensed and diluted phases. This temperature induced behaviour is likely to be entropically-driven, and determined by the IDR conformational ensemble at moderate temperature, leading to a temperature-induced expansion when IDRs behave as random coils and/or are typically collapsed at low/moderate $T^{*}$ (due to intra-molecular interactions), and to entropically-driven collapse when IDRs are moderately extended at low/moderate temperature (due to the high energetic bending penalty). Consistently, while IDRs are commonly ascribed as random coils and are expected to expand monotonically with increasing temperature [110], confocal single-molecule FRET experiments showed that some IDRs at infinite dilution can also exhibit temperature-induced collapse [111]. Cold-shock proteins are one example of IDRs that collapse with temperature [112]. Although the hydrophobic character of IDRs is a contributing factor to their temperature-induced collapse, a subtle interplay between the intra-protein and protein-solvent interactions can also lead hydrophilic IDRs to present this behaviour [112,113]. Our CG model predicts that IDRs exhibiting temperature-induced expansion may have an advantage to phase separate, over those presenting temperature-induced collapse (Figure 3C), because their expansion decreases the steric barrier for establishing inter-protein interactions (see Table 1). However, irrespectively of their temperature-dependent behaviour, IDRs with a higher radius of gyration at a given conditions (i.e., temperature), show higher ability to establish LLPS-stabilising protein–protein interactions, and thus greater ability to phase separate (see Table 1 and Figure 3B).

**Figure 5:**
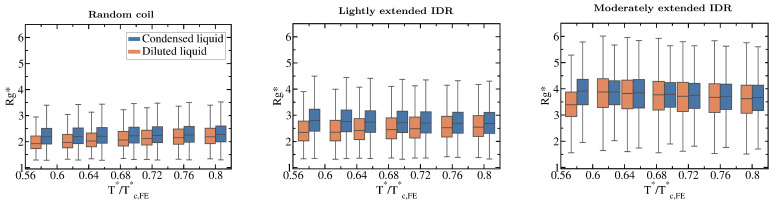
Impact of the liquid–liquid phase transition on the protein conformational ensemble. Boxplots of the radius of gyration vs. normalised temperature ($T^{*}/T_{c,FE}^{*}$) for the fully flexible random coil, and the lightly and moderately extended N = 20 IDRs. Orange boxplots account for $R_{g}^{*}$ in the diluted phase while blue ones for IDRs in the condensed phase. Fully extended IDR boxplots have not been included since $R_{g}^{*}$ is not different in both phases (according to the Kolmogorov-Smirnov test [115]) and does not vary with temperature either as shown in Figure 3C. Boxplots are a 5 number summary of the radius of gyration distribution. The box bounds represent the first and the third quartile of the histograms and the line intersecting the box is the median. The whiskers represent the maximum and minimum values of the histograms.

Our simulations also reveal how the conformational ensemble of IDRs can be affected by the liquid–liquid phase transition. When IDRs undergo homotypically driven LLPS, the distributions of their radii of gyration remain virtually unchanged. Although fully flexible and lightly extended IDRs exhibit a modest phase-separation driven expansion, such expansion occurs only at low to moderate temperatures ($T^{*}<0.75T_{c}^{*}$, where $T_{c}^{*}$ refers to the critical temperature of each IDR). As we approach the critical temperature, the radii of gyration of the proteins among the two phases become increasingly similar (see Boxplots in Figure 5). Phase-separation driven expansion for proteins undergoing homotypic LLPS was observed for tau-IDP [114] using steady-state fluorescence measurements of pyrene and fluorescein-labeled tau-K18 proteins, a protein associated with Alzheimer’s disease. Even if modest, phase-separation induced expansion enables IDRs to establish a surplus of enthalpy maximizing inter-protein contacts in the condensed phase than those they would establish if they remained unchanged or underwent collapse.

### 2.4. Collapsed Globular Domains Inhibit LLPS

We now compare the phase diagrams of flexible IDRs with those of proteins that have a rigid globular structure but the same exact amino acid sequence and inter-bead binding strength. We compare two distinct configurations of a 20-bead globular protein with different degrees of compaction: (1) a semi-compact structure with a radius of gyration of 2.40 (in reduced units), and (2) a compact structure with radius of gyration of 1.75.

**Figure 6:**
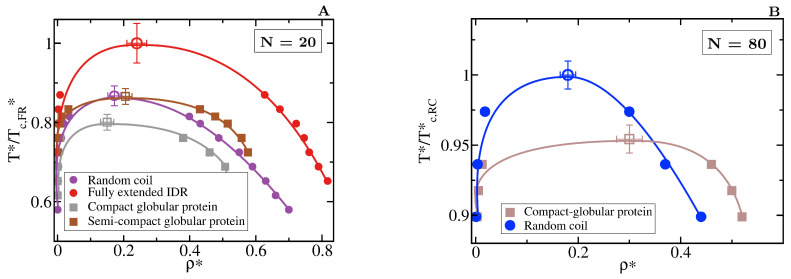
Role of globular and compact conformations in LLPS. (**A**) Phase diagram of different conformational ensembles of the 20-bead sequence. Results for the random coil are depicted in purple, for the fully extended IDR in red, while for the semi-compact and compact globular proteins in brown and grey, respectively. Filled circles represent the liquid coexisting densities evaluated by means of DC simulations, empty symbols the estimated critical point for each system via Equations (4) and (5), and continuous lines are included as a guide for the eye. (**B**) Phase diagram of an 80-bead fully flexible random coil with $<R_{g}>$ = 4.42σ (at $T^{*}/T_{c,RC}^{*}=0.9$) (blue) and a collapsed globular conformation with $<R_{g}>$ = 2.45σ (red) of length N = 80. Note that in both panels, temperature is normalised by the critical temperature of the conformational ensemble with highest critical point for each length, $T_{c,FE}^{*}$ = 2.76 for the fully extended 20-bead IDR and $T_{c,RC}^{*}$ = 2.68 for the random coil of 80 beads. The structured globular domains for which the phase diagram is evaluated here are shown in Figure 1.

The specific conformation that a protein adopts has a crucial effect on its liquid–liquid phase behaviour. As shown in Figure 6A, IDRs that predominantly display extended conformations across a relevant temperature range for LLPS, such as the fully extended IDR (red curve), phase separate up to significantly higher temperatures. Conversely, more collapsed globular domains or IDRs that maintain smaller radii of gyration show a lower propensity to phase separate. Such dramatic effect of the structural behaviour of proteins in their phase landscape is again explained through the molecular connectivity (Figure 4). Given the same protein sequence, and assuming that the binding affinity between amino acids is unchanged among the different protein configurations, the number of molecular contacts that extended IDRs can establish is usually higher than those enabled by a more compact structure that buries the LLPS-stabilizing binding sites deep within (Figure 4). Interestingly, we note that below a certain number of contacts ∼15 (contacts per protein for the 20-bead sequences) LLPS vanishes. By re-scaling the phase diagrams in $T^{*}/T_{c}^{*}$ vs. $\rho^{*}$ (being $T_{c}$ the critical temperature of each protein), we observe general scaling, where the minimum amount of inter-molecular contacts corresponds to a reduced density of ∼0.35, below which, LLPS is not stable. The correlation between the mean radius of gyration, end-to-end distance, average number of inter and intra-protein contacts, and liquid–liquid critical point is reported in Table 1.

If we now compare larger proteins, we observe that the impact of the structural conformation in the phase behaviour is moderately amplified. In Figure 6B, we compare the phase diagram of an 80-bead random coil with that of an 80-bead compact globular protein of equal sequence. We observe that when all other factors are kept equal, the critical temperature of the random coil is considerably higher than that of the globular protein. Again, the molecular connectivity evaluated by the number of inter-molecular contacts for the random coil is more than twice than for the globular conformation (see Table 1). Such difference is expected to increase if the conformational ensemble of the IDR is further enriched in extended configurations. In longer sequences, the amount of buried amino acids is larger because of the surface to volume ratio scaling, as shown in Table 1, which imposes steric hindrance to establish inter-protein interactions. Recent experiments on G3BP1, a protein involved in the formation of stress granules which presents RNA-mediated conformational switching, highlights the crucial role of protein conformation in liquid–liquid phase separation [116]. Under non-stress conditions, G3BP1 dimers adopt an autoinhibited highly compact state that reduces the available number of inter-molecular interactions that the protein can establish [116]. However, upon stress, released mRNA outcompetes for the G3BP1 compacting intra-molecular interactions and induces expansion of G3BP1, which in turn, promotes LLPS of the G3BP1 and RNA mixture [116]; this can be explained by an enhanced ability of G3BP1 to establish multivalent heterotypic protein-RNA and homotypic intermolecular protein–protein interactions. Even though the expansion of G3BP1 is RNA-mediated, and the LLPS enhancement is coupled to the enthalpic contribution through protein-RNA interactions, our CG simulations suggest that the conformational expansion itself plays a big role in promoting LLPS of the G3BP1-RNA mixture.

### 2.5. Influence of the Conformational Ensemble in Heterotypically Driven LLPS

A significant number of intrinsically disordered proteins exhibit heterotypically driven phase separation, i.e., dependent on their binding to a cognate protein or RNA [117]. For example, P bodies fundamentally assemble via interactions between RNA-binding proteins and RNA [33,118]. Heterotypic interactions among proteins and RNA components [119] contribute to the formation of other intracellular condensates such as Cajal bodies and stress granules. The nucleolus, a phase separated organelle within the nucleus, is stabilized by a combination of heterotypic and homotypic interactions among the Nucleophosmin (NPM1) RNA-binding protein and ribosomal RNA [120,121]. To consider heterotypically driven phase separation, we focus on 50:50 binary mixtures of two types of IDRs, that we term protein A and protein B. IDRs of type A interact most strongly with IDRs of type B and vice-versa, whereas IDRs of the same type exhibit smaller binding affinity (i.e., the well-depth of the heterotypic LJ interaction $\epsilon_{He}$ is double than the homotypic LJ one $\epsilon_{Ho}$, as sketched in Figure 1). Hence, systems exclusively composed of a single type of IDRs (A or B) can only phase separate at very low temperatures.

**Figure 7:**
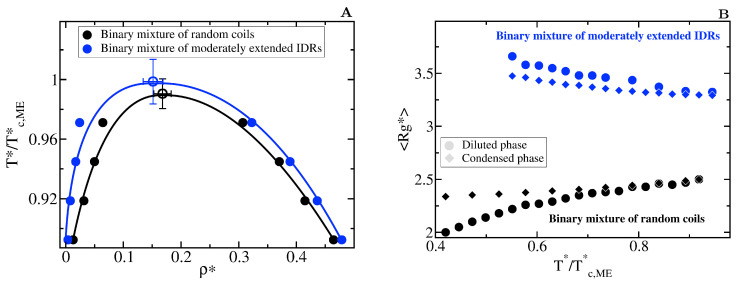
Impact of the conformational plasticity in heterotypically driven LLPS (**A**) Liquid-liquid coexistence lines of two 50:50 binary mixtures of 20-bead IDRs with different conformational ensembles: filled black circles represent the coexistence densities for a mixture of fully flexible random coils while blue circles for a mixture of moderately extended IDRs. Empty circles indicate the estimated critical point of each mixture. Note that temperature is renormalized by the critical temperature of the moderately extended IDR binary mixture, $T_{c,ME}^{*}$ = 3.8. (**B**) Average radius of gyration, $<R_{g}^{*}>$ of the IDRs as a function of the renormalized temperature, $T^{*}/T_{c,ME}^{*}$. Circles indicate $<R_{g}^{*}>$ in the diluted liquid phase and diamonds inside the condensed one. The same colour code of the aside panel applies here.

We model two binary mixtures of 20-bead IDRs with different conformational ensembles: a mixture where both types of IDRs behave as fully flexible random coils ($K_{\theta }^{*}$ = 0) and a mixture of moderately extended IDRs ($K_{\theta }^{*}$ = 3). Extended states also favour heterotypically driven LLPS (Figure 7A). However, the behaviour of the radius of gyration for the moderately extended IDRs along the phase transition (Figure 7B) differs from the one observed in homotypically driven LLPS. Such moderately extended IDRs become more compact after undergoing heterotypically driven phase separation. Given that the intra-molecular self-interactions in heterotypically driven phase separation are weak, IDRs restricted to sample more extended states have a negligible enthalpic gain for collapsing and, hence, remain extended in the diluted phase. Upon condensation, binding to cognate partners facilitates moderate compaction. Consistent with our previous observations, the mildly self-interacting IDRs that we study in this section undergo temperature-induce expansion when they are fully flexible, and temperature-induced collapse when they are restricted to sample extended configurations.

Hence, our results highlight that irrespective of whether LLPS is sustained by homotypical or heterotypical interactions, switching the conformational ensemble of an IDR to one enriched in extended states, unambiguously promotes liquid–liquid phase separation by enhancing the protein molecular connectivity.

## 3. Materials and Methods

### 3.1. Direct Coexistence Simulations

To compute the phase diagram of the different protein systems, we perform Direct coexistence (DC) simulations [103,104,105]. Such method (see Figure 2) consists of simulating periodically extended slabs of the two coexisting phases, e.g., the diluted and the condensed phases, in the same simulation box. To evaluate the density of the coexisting phases, once the system is equilibrated, we compute a density profile along the long side of the box (perpendicular axis to the interface) and average the density of each phase, excluding the density fluctuations near the interface. Due to finite size effects, it is not computationally feasible to determine the critical point using the DC method. Hence, we estimate the critical density and temperature of the phase diagrams by fitting the coexisting densities of both phases near the critical point using the laws of rectilinear diameters and critical exponents [94,122]:

(4) $${\left(\rho_{h}^{*}-\rho_{l}^{*}\right)}^{\alpha }=s_{1}\left(1-T^{*}/T_{c}^{*}\right)$$

(5) $$\left(\rho_{h}^{*}+\rho_{l}^{*}\right)/2=\rho_{c}^{*}+s_{2}\left(T_{c}^{*}-T^{*}\right)$$

where $\rho_{h}^{*}$ and $\rho_{l}^{*}$ account for the coexisting (reduced) densities of the protein-rich liquid phase and the protein-poor liquid one respectively, $T_{c}^{*}$ and $\rho_{c}^{*}$ are the critical temperature and density respectively, α=3.06 is the three dimensional Ising model critical exponent [122], and s1 and s2 are fitting parameters.

### 3.2. Liquid Network Connectivity

To quantify the number of molecular connections per protein replica, or liquid network connectivity within the condensates, we use the following procedure [10]. For the protein-rich liquid phase (condensed liquid), we prepare a system in the NVT ensemble at the coexisting density and temperature. For each individual protein, we count the number of beads of other proteins (i.e., excluding the intra-molecular contacts since those are not involved in sustaining the condensate) that are closer than a cut-off distance of 1.2σ. We verified that the connectivity trends discussed in this work persist regardless of the chosen cut-off distance. Within the protein-poor liquid phase (diluted phase), densities are rather small and inter-molecular contacts scarce. Thus, for the diluted phase, we count the number of intra-molecular contacts instead, which is an indirect measure of the degree of compaction of the IDRs in isolation. The same cut-off distance of 1.2σ is used.

## 4. Conclusions

In this work, we investigate the impact of a protein conformational landscape in its liquid–liquid phase behaviour. Using a minimal coarse-grained model, we find that enriching the conformational ensemble of an IDR in extended configurations significantly promotes LLPS. Conversely, switching to an alternate configurational ensemble that predominantly samples compact (rigid or flexible) conformations inhibits LLPS, provided that such a structural change does not alter the binding strength among proteins.

Our simulations reveal that the molecular origin of LLPS enhancement stemming from enriching an IDR conformational ensemble in extended structures is the increase in the molecular connectivity of the condensed liquid [10]. Extended protein conformations facilitate LLPS-stabilising protein–protein interactions by reducing the steric hindrance, and thereby maximising the molecular connectivity of the liquid network. This behaviour holds regardless of whether phase separation is driven by homotypic and/or heterotypic molecular interactions.

We find that, within our model, fully flexible random coils and lightly extended IDRs undergo temperature-induced expansion, while IDRs more significantly constrained to sample extended states exhibit moderate temperature-induced collapse. Even though IDRs that exhibit temperature-induced expansion may have an advantage to phase separate over those exhibiting temperature-induced collapse, because of a surplus of inter-protein interactions, our CG simulations show that IDRs with a higher radius of gyration at a given temperature, irrespectively of their temperature-dependent behaviour, will have a greater ability to phase separate.

Furthermore, we show that the conformational landscape of IDRs is lightly altered upon the liquid–liquid phase transition, with the more notable changes emerging at lower temperatures and higher protein–protein binding affinities. Either in homotypically or heterotypically driven LLPS, highly flexible IDRs adopt slightly more expanded conformations when transitioning from the diluted phase to the condensed phase. However, in heterotypically driven LLPS, moderately extended IDRs become more compact upon phase separation. Together this work contributes to advancing our understanding of the relationship between protein conformation and liquid–liquid phase behaviour.

## Figures and Tables

**Table 1:** Structural conformation, molecular contacts and thermodynamic stability of the protein condensates. Average radius of gyration ($<R_{g}^{*}>$), end-to-end distance ($<D_{E-E}>$) and number of intra-molecular contacts per protein ($N_{Intra}^{C}$) in the diluted phase; number of inter-molecular contacts per protein in the condensed phase ($N_{Inter}^{C}$) and critical temperature ($T_{c}^{*}$) of the different studied proteins. The given values of $<R_{g}^{*}>$, $<D_{E-E}>$, $N_{Intra}^{C}$ and $N_{Inter}^{C}$ for the 20-bead (80-bead) proteins correspond to a temperature of $T^{*}=0.72T_{c,FE}^{*}$ ($T^{*}=0.9T_{c,RC}^{*}$) and to the density of the coexisting phase of interest at such temperature. $T_{c,FE}^{*}$ = 2.76 and $T_{c,RC}^{*}$ = 2.68. Please note that the radius of gyration and the number of intra-molecular contacts for the globular structured proteins are temperature-independent.

N	Protein Conformation	$<R_{g}^{*}>$	$<D_{E-E}>$	$N_{\mathrm{Intra}}^{C}$ Dil. Phase	$N_{\mathrm{Inter}}^{C}$ Cond. Phase	$T_{c}^{*}$
20	Fully extended IDR	5.49	17.14	0.0	42	2.76
20	Moderately extended IDR	3.71	10.12	0.3	32	2.42
20	Lightly extended IDR	2.53	6.15	2.0	27	2.37
20	Random coil	2.14	5.34	3.4	23	2.34
20	Semi-compact globular protein	2.40	6.64	2.0	23	2.34
20	Compact globular protein	1.75	4.18	5.0	16	2.21
80	Random coil	4.42	10.54	18	51	2.68
80	Compact globular protein	2.45	7.84	63	20	2.54
